# Wafer-Level Vacuum-Packaged Translatory MEMS Actuator with Large Stroke for NIR-FT Spectrometers

**DOI:** 10.3390/mi11100883

**Published:** 2020-09-23

**Authors:** Thilo Sandner, Eric Gaumont, Thomas Graßhoff, Andreas Rieck, Tobias Seifert, Gerald Auböck, Jan Grahmann

**Affiliations:** 1 Fraunhofer Institute for Photonic Microsystems (FhG-IPMS), 01109 Dresden, Germany; eric.gaumont@ipms.fraunhofer.de (E.G.); thomas.grasshoff@ipms.fraunhofer.de (T.G.); andreas.rieck@ipms.fraunhofer.de (A.R.); jan.grahmann@ipms.fraunhofer.de (J.G.); 2 Fraunhofer Institute for Electronic Nanosystems (FhG-ENAS), 09126 Chemnitz, Germany; tobias.seifert@enas.fraunhofer.de; 3 Silicon Austria Labs (SAL), 9524 Villach, Austria; gerald.auboeck@silicon-austria.com

**Keywords:** micro-opto-electro-mechanical system (MOEMS), translatory micro mirror, optical wafer-level vacuum package (WLVP), NIR Fourier-transform spectrometer, glass-frit wafer bonding

## Abstract

We present a wafer-level vacuum-packaged (WLVP) translatory micro-electro-mechanical system (MEMS) actuator developed for a compact near-infrared-Fourier transform spectrometer (NIR-FTS) with 800–2500 nm spectral bandwidth and signal-nose-ratio (SNR) > 1000 in the smaller bandwidth range (1200–2500 nm) for 1 s measuring time. Although monolithic, highly miniaturized MEMS NIR-FTSs exist today, we follow a classical optical FT instrumentation using a resonant MEMS mirror of 5 mm diameter with precise out-of-plane translatory oscillation for optical path-length modulation. Compared to highly miniaturized MEMS NIR-FTS, the present concept features higher optical throughput and resolution, as well as mechanical robustness and insensitivity to vibration and mechanical shock, compared to conventional FTS mirror drives. The large-stroke MEMS design uses a fully symmetrical four-pantograph suspension, avoiding problems with tilting and parasitic modes. Due to significant gas damping, a permanent vacuum of ≤3.21 Pa is required. Therefore, an MEMS design with WLVP optimization for the NIR spectral range with minimized static and dynamic mirror deformation of ≤100 nm was developed. For hermetic sealing, glass-frit bonding at elevated process temperatures of 430–440 °C was used to ensure compatibility with a qualified MEMS processes. Finally, a WLVP MEMS with a vacuum pressure of ≤0.15 Pa and *Q* ≥ 38,600 was realized, resulting in a stroke of 700 µm at 267 Hz for driving at 4 V in parametric resonance. The long-term stability of the 0.2 Pa interior vacuum was successfully tested using a Ne fine-leakage test and resulted in an estimated lifetime of >10 years. This meets the requirements of a compact NIR-FTS.

## 1. Introduction

Near-infrared spectroscopy (NIRS) with spectral wideband light sources is an extremely versatile method used for quality control in chemical production processes. As absorption bands in the near-infrared spectral range (800–2500 nm) are relatively weak, the method can be used for an analysis of a wide range of materials in very diverse states (gas, liquid, and solid, as well as heterogeneous multicomponent systems) [1]. NIRS is used, e.g., to determine water in plastics or for rapid, nondestructive moisture measurement in food quality control or for physical parameters such as viscosity or grain size. Practically, quantitative chemical analysis using NIRS relies on multivariate statistical analysis using a prediction model built from spectra of calibration samples for which the analyte is known from primary reference measurements (such as Karl Fischer titration for the case of water [2]). Typically, NIR spectra in condensed phases contain very broad, overlapping bands. In order to capture a sufficient number of spectral bands for a reliable analytical result, NIR spectrometers are, thus, equipped with wideband sources. Such sources are realized by black-body emitters such as tungsten halogen lamps. As they have a low brightness due to the undirected thermal emission, the design of an NIR spectrometer involves optimizing the coupling of the source into the spectrometer and its optical throughput.

### 1.1. MEMS-Based Fourier-Transform (FT) Spectrometer

As the Fourier-transform (FT) spectrometer based on the Michelson interferometer offers the highest throughput due to the multiplexing and etendue advantage over dispersive spectrometers [1,3], it has found numerous industrial applications so far. Conventional FT spectrometers do, however, suffer from slow acquisition (compared to diode-array-based dispersive spectrometers [4]) due to the inertia of the path-length scanning optics. For rough environments (in terms of vibration and shock) typically encountered in chemical process applications, the required mechanical stability implies that the scanning mechanics of an FT spectrometer is relatively bulky compared to diode-array spectrometers.

MOEMS (micro-opto-electromechanical systems) have been investigated during the last decade for robust and fast optical path-length modulation, miniaturization, and cost reduction of FT spectrometers. They have the potential of making FT-NIRS a candidate technology for efficient, cost-effective spectroscopy solutions for industrial chemical analytics. Various MEMS concepts for optical path-length scanning have been reported, typically targeting an out-of-plane translation with large stroke required for the classical dual-beam Michelson interferometer FTS set-up, consisting of fixed and moving mirrors (here realized by an MOEMS) and an optical beam splitter. For large-stroke out-of-plane translation of the MEMS mirror, different actuation principles have been investigated so far: electrostatic [5,6,7,8], piezoelectric [9,10,11], magnetic [12,13,14,15], and electrothermal [16,17,18,19,20,21,22], typically using resonant operation for larger strokes, e.g., [7,8,11], but also using quasi-static actuation, e.g., [10,17]. Most of these different MOEMS actuation mechanisms suffer from limitations in spectral resolution due to parasitic effects of MEMS-based path-length modulation: first of all, mirror tilt [11,17,19], deformation of the mirror due to dynamically (owing to inertia) or statically (due to mirror suspension or optical coating) induced mechanical stress.

In addition to the actuation mechanism, MEMS-based FTS can be subclassified by the principle of optical FTS instrumentation: (i) Michelson interferometer-based FTS, e.g., [7,12,23,24,25,26,27], or (ii) lamellar grating-based FTS, enabling a simplified interferometer set-up without optical beam splitter [6,8,28,29,30,31,32]. MEMS technology was also used to simplify the optical system integration of FTS; early approaches using bulk [5,6,33] and surface micromachining [34] were reported. During the last decade, monolithic integration of the interferometer optical bench into silicon was realized for a higher miniaturization and cost reduction of MEMS-based FTS, e.g., [35,36,37,38]. Although full on-chip integration of FT interferometers (including the translatory MOEMS) is limited to small mirror apertures and imperfect surface quality, these MEMS-FT spectrometers are starting to enter the market [38] for NIR applications and are used in cases with moderate requirements of spectral resolution due to their advantages in size and cost.

**Figure 1:**
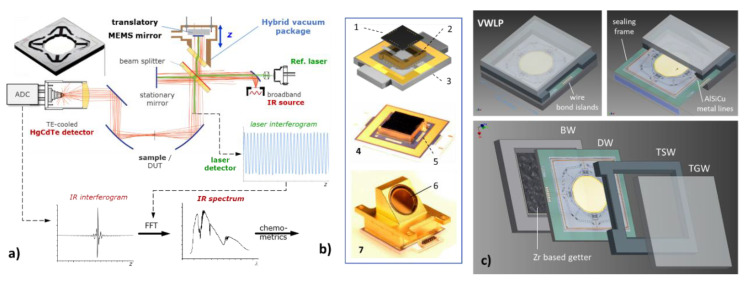
MEMS-based IR-FTS using a large-stroke four-pantograph MEMS mirror: (**a**) schematic optical FTS set-up designed for a broad MIR (mid-infrared) spectral range of 2.5…16 µm using a translatory MEMS [27]; (**b**) hybrid optical vacuum package of MEMS using a ZnSe window due to broad spectral MIR range [25]: 1, MEMS chip; 2, cover spacer; 3, ceramic board; 4, MEMS chip soldered on ceramic board; 5, Al wire bonds; 6, ZnSe window with broad-band anti-reflex coating (BB-ARC); 7, final hybrid vacuum package; BW, bottom wafer; DW, device wafer; TSW, top spacer wafer; TGW, top glass wafer); (**c**) wafer-level vacuum package (WLVP) of MEMS for NIR-FTS with a spectral range of 0.8–2.5 µm.

Our work on compact MEMS IR-FTS is based on a classical Michelson FTS instrumentation, but using electrostatic resonant MEMS mirrors with large strokes for fast optical path-length modulation [7,23,24,25,26,27]. This MEMS-FTS approach, combining a classical optical instrumentation with a resonant MEMS (see Figure 1a), enables larger mirror apertures (required for high optical throughput), a larger stroke of translatory oscillation of up to 1.4 mm [25], and an extreme repeatability and stability of the optical path modulation due to high *Q* resonant operation. These are advantages over monolithic MEMS-FTS and enable robust compact FT spectrometers with improved spectral resolution and SNR (signal-noise-ration). On the other hand, due to the significant gas damping [25], the resonant MEMS mirror has to operate in vacuum to achieve large strokes. In the past, we developed translatory MEMS mirrors with large strokes for a broadband MIR-FTS with a spectral range of *λ* = (2–16) µm. In [26], a spectral resolution of *∆ν* = 12 cm^−1^ (4.5 nm @ 2 µm) and an SNR ≥ 1000:1 (for ≥200 ms measuring time and co-addition of spectra) were verified by experiment. In a single scan, an SNR of 48 was achieved, while averaging 500 scans improved the SNR to 1607. The translatory MEMS device uses a point-symmetric pantograph suspension of the 5 mm mirror [25]. To achieve the full scan amplitude of 500 μm at 500 Hz, a vacuum pressure of ≤10 Pa was required. The MIR-FTS previously reported in [26,27] required a high optical MIR transmission without spectral gaps over the entire MIR spectral range. For that reason, a costly hybrid assembly with a broadband ARC-IR window of ZnSe had to be chosen for MEMS vacuum packaging (see Figure 1b, [25]), instead of a cost-effective wafer-level vacuum package, e.g., using a silicon or glass window.

### 1.2. Motivation of This Study

In this article, we present a wafer-level vacuum-packaged translatory MOEMS device, especially developed for a compact NIR-FT spectrometer operating in the NIR spectral range *λ* = (800–2500) nm. The MEMS-based NIR-FTS targets a spectral resolution ≤15 cm^−1^ and SNR >1000 for a measuring time of 1 s. For a fast optical path-length modulation, it requires a highly precise (tilt-free) out-of-plane MEMS translation with 350 µm amplitude (700 µm stroke) and only 80 nm dynamic mirror deformation. Although we developed a translatory (pantograph) MEMS with 500 µm scan amplitude for MIR-FTS [25], it is not suitable for an NIR-FTS with *λ_min_* = 800 nm due to the significantly too large dynamic mirror deformation of *δ*_p-p_ = 433 nm. Even for a reduced amplitude of 350 µm, this MEMS has a too large dynamic mirror deformation of *δ*_p-p_ = 303.1 nm (*λ_min_*/2.6), which exceeds the specified value of 80 nm by a factor of 3.8. Hence, no pantograph MEMS for an NIR-FTS exists so far (see Table 1). To achieve the specified amplitude of 350 µm, the translatory MEMS has to be encapsulated in an optical vacuum package as well. Due to the NIR spectral range, a glass window with broadband antireflective coating (BB-ARC) is applicable instead of the zinc selenide (ZnSe) used in [25]. Now, vacuum MEMS packaging can be performed at wafer level instead of the previous cost-intensive hybrid package.

In this work, we contribute new results concerning the following three main topics:

- NIR-FTS-specific design of a resonant large-stroke pantograph MEMS device with minimized dynamic mirror deformation of *δ*_p-p_ = 80 nm at 350 µm scan amplitude,
- development of a cost-effective optical (NIR) wafer-level vacuum package with process compatibility to the existing qualified MEMS scanner process AME75 of Fraunhofer IPMS,
- detailed characterization of new translatory MEMS devices with WLVP, investigation and elimination of failure mechanisms of WLVP (e.g., influence of process temperature on mirror planarity), and long-term stability of the desired vacuum pressure for ≥10 years lifetime.

**Figure 2:**
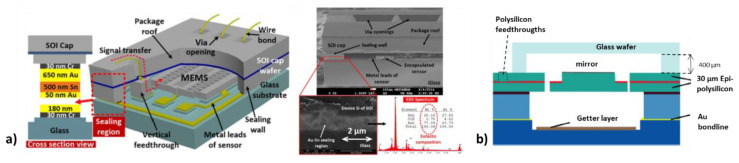
Examples of state-of-the-art wafer-level MEMS vacuum packages: (**a**) WLVP of a MEMS resonator using alternating layers of evaporated Au and Sn [39]; (**b**) schematic set-up of wafer-level vacuum-packaged micro scanning mirrors using anodic bonding of a glass cover wafer to a polished Epipoly sealing frame and eutectic AuSn bonding of the bottom wafer to the MEMS backside [40].

A broad variety of wafer-level vacuum-packaging technologies and wafer bond techniques exists for hermetic sealing of smart MEMS sensors [39] or MOEMS devices, e.g., torsional MEMS mirrors [40,41,42,43]. Two examples of wafer-level MEMS vacuum packages are exemplarily shown in Figure 2. Typically, MEMS WLVPs have a closed sealing area with low topography for hermetic wafer bonding. Vertical feedthroughs are also often used for electrical signal transfer from the inner cavity of WLVP.

Concerning the wafer-level-packaging of micro scanning mirrors, we mention the state of the art described in [40,41,42,43]. Here, a micro molded glass cap wafer with 400–900 µm cavity height is anodically bonded to the polished surface of an epi-polysilicon MEMS device [40,41]. The MEMS backside is hermetically sealed by eutectic Au bonding [44] using a 3 µm thick electroplated Au layer. Using getters, a vacuum pressure of 0.1 mbar (10 Pa) was achieved in [40]. In [42], a modified WLVP process of micro scanning mirrors was presented, which allows tilted glass windows on a wafer level (instead of a parallel window). For hermetic sealing of the top glass wafer, glass-frit bonding was now used instead of anodic bonding (enabling a free choice of glass material and avoiding high process voltages). In [42], an inner vacuum pressure of 0.1 Pa was reported using a titanium thin-film getter. Furthermore, a modular packaging concept for MOEMS was presented in [43] allowing also vacuum packages with integrated vertical electrical feedthroughs (through-glass via, TGA).

The concept of the MEMS WLVP reported in this work (see Figure 1c) was determined by the following specifics and limitations of the qualified MEMS scanner process AME75 [45]:

- AlSiCu, which is used for metal signal lines and bond island45s (at the outer chip frame),
- high topography (≥2 µm due to metal lines) within the areas needed for hermetic sealing,
- ultrasonic Al wire bonding required for good electrical contact of AlSiCu,
- use of filled isolation trenches for electrostatic comb drives (also adding surface topography),
- CMOS compatibility of all in-line processes used for fabrication of MEMS device wafers.

Due to the fixed MEMS process, the following consequences result for the NIR-WLVP: (i) no area with low topology exists on the MEMS surface (sealing frame is crossed by metal lines, see Figure 1c) and (ii) vertical signal feedthroughs are not applicable due to the poor electrical contact to the AlSiCu surface. Hence, a hermetic bond technique is required which can tolerate high surface topologies of ≥2 µm. For this NIR-WLVP application, glass-frit bonding (which requires elevated process temperatures of 430–440 °C) was selected to be the best sealing method [46,47,48,49,50,51,52] in order to remain compatible with the fixed MEMS scanner process (see Table 2 for a comparison of alternative hermetic bonding techniques). Earlier work on glass-frit-bonded WLVP of AME75 processed MEMS mirrors resulted in 500 Pa internal pressure [52] (without getter) (in this work, <1 Pa was required). For similar MEMS mirrors, a significant degradation of mirror planarity was observed at elevated process temperatures. The radius of mirror curvature decreased below *R* < 2 m at temperatures above 350 °C [53] (equal to a mirror deformation of *δ_pp_* > 1.56 µm assuming a 5 mm mirror diameter), which was not acceptable for this work. An initial concept for NIR-MEMS WLVP using glass-frit bonding and integration of a state-of-the-art Zr-based getter for long-term stabilization of the internal pressure below <1 Pa was presented in [54]. Very first results demonstrate exemplarily a sufficient inner vacuum pressure of 0.25 Pa using only a single WLVP stack with simplified set-up (without Au mirror coating, no BB-ARC). New experimental results using a complete WLVP set-up (with Au mirror coating and BB-ARC) show that the initial NIR-WLVP concept [54] failed due to reliability issues. The main failure mechanism is unknown (e.g., inner outgassing sources, degradation of the Au coating).

In this article, we carefully investigated the influence of technological factors (e.g., elevated process temperature during glass-frit bonding) on the inner vacuum pressure, optical performance (e.g., static mirror deformation), and long-term stability of the MEMS WLVP. The degradation of Au mirror planarity at elevated process temperatures (up to 440 °C) was observed as the main failure mechanism. Potential failure sources for outgassing and degradation of the long-term stability of the inner vacuum pressure were also investigated. These reliability issues were eliminated within the modified WLVP process, e.g., by using an additional Al_2_O_3_ diffusion barrier layer to prevent thermally induced degradation of the Au mirror coating. Finally, a translatory MEMS WLVP with vacuum pressure ≤0.25 Pa and Q > 18,000 was realized, resulting in a stroke of 700 µm at 267 Hz for driving at 4 V in parametric resonance. The long-term stability of the 0.2 Pa inner vacuum was successfully tested, which was estimated to be >10 years using a Ne fine-leakage test. Parasitic tilt of 20 arcsec was measured for selected MEMS devices over the full 700 µm scan. These meet the requirements of a compact NIR-FTS with high SNR > 1000 and a spectral resolution of ∆ν ≤ 15 cm^−1^ for a spectral bandwidth of 1200–2500 nm.

In this article, we show that glass-frit bonding can be used for a relatively simple and cost-effective WLVP process of optical MEMS, while avoiding degradation of optical performance even at high process temperatures of up to 440 °C. In addition to the NIR-FTS application, our experimental results and the observed failure mechanism of the glass-frit based WLVP process reported in this work could be helpful to a broader community using glass-frit bonding for low-cost WLVP of various smart MEMS sensors or MOEMS applications.

## 2. Materials and Methods

This translatory MEMS actuator with optical WLVP was especially developed for fast optical path-length modulation in a compact NIR-FT spectrometer for NIR spectral region λ = 800–2500 nm. The general specification of NIR-FTS and boundary conditions (considered for the MEMS design) are summarized in Table 2. Additional requirements follow from the used MEMS processes.

In this work, we reduced the scan amplitude of a 5 mm mirror aperture from 500 µm to 350 µm in comparison to the previous MEMS-based MIR-FTS [25]. With a stroke of 700 µm (equal to twice the amplitude), this MEMS provides a spectral resolution of ∆ν = 14.2 cm^−1^ for a similar FTS instrumentation (shown in Figure 1a). This is useful, because requirements on parasitic MEMS properties, e.g., parasitic tilt and static and dynamic mirror deformation, are significantly higher due to the smaller wavelength. This can be seen in comparison to the requirements for a conservative NIR-FTS design, targeting a high spectral resolution of ∆ν = 8 cm^−1^. A very small value of only 2” follows for the parasitic tilt angle, which has to be guaranteed over the entire stroke of 1.25 mm; this would be highly risky. Moreover, a small mirror deformation of 80 nm = 1/10 λ_min_ (p-p value) results from the minimal wavelength of 800 nm. Both (i) parasitic mirror tilt and (ii) static mirror deformation are the main challenges for the MEMS and WLVP process due to (i) geometrical tolerances of narrow spring geometries caused by the deep reactive ion etching (DRIE) process, and (ii) static mirror deformation resulting from thermal stress on the optical coating induced by high WLVP process temperatures of up to 430–440 °C, required for hermetic glass-frit bonding.

### 2.1. Translatory MEMS Design

During the MEMS design process, the following technological constraints were fixed by the MEMS scanner technology, available at FhG-IPMS [45]:

- electrostatic resonant driving using vertical comb drives,
- use of 75 µm thick SOI (silicon-on-isolator) layer of monocrystalline silicon,
- no additional stiffening structures at mirrors backside.

The main challenges for this MEMS are (i) to enable a tilt-free large stroke with (ii) reduced static or dynamic mirror deformation and (iii) high mechanical reliability. An early design approach for translatory MEMS with 1.65 mm^2^ aperture was based on a mirror suspension using two folded bending springs, resulting in a limited amplitude of 100 µm and significant dynamic mirror deformations caused by the mirror suspension itself [7]. Next, in [23], a 1 kHz translatory 3 mm mirror with two pantograph suspensions was tested to increase the scan amplitude to 300 µm. Here, the pantograph suspensions use torsional springs as deflectable elements instead of bending springs. This has the potential for larger deflection and reduced mechanical stresses coupled into the mirror plate at the same time. Unfortunately, due to superimposed parasitic torsional modes, only an amplitude of 140 µm could be measured for the two-pantograph MEMS device, which is not suitable for an FTS. The problem of mode separation was fixed in [25] for a translatory MEMS device with 500 Hz and 5 mm diameter mirror using a fully symmetric mechanical design of four pantograph suspensions, enabling large strokes of up to 1.4 mm and avoiding problems with parasitic modes and tilting.

#### 2.1.1. Pantograph Mirror Suspension for Large Stroke

**Figure 3:**
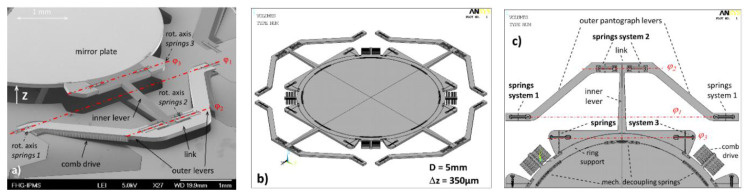
Pantograph mirror suspension for large-stroke translatory MEMS: (**a**) SEM detail of single pantograph (pre-deflected, three parallel torsional spring axes are obvious); (**b**) translatory MEMS device designed for NIR with full symmetric suspension of 5 mm mirror using four pantographs (FEA geometry model of movable elements); (**c**) details of pantograph suspension of NIR-MEMS design.

**Figure 4:**
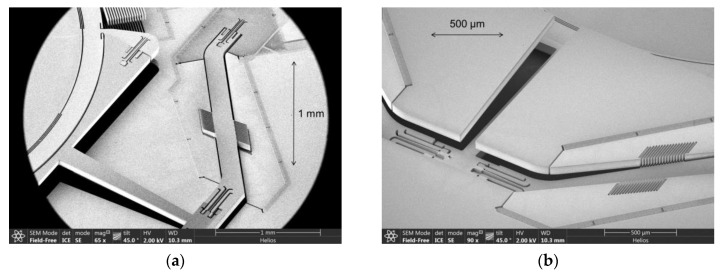
SEM details of single pantograph suspension: mirror moved (**a**) up and (**b**) down.

**Figure 5:**
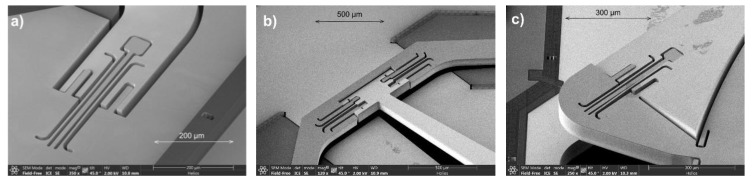
SEM pantograph details of deflectable spring system: (**a**) spring 1; (**b**) spring 2; (**c**) spring 3.

The actual NIR-FTS optimized translatory MEMS device uses also a point-symmetric configuration of four pantograph suspensions of a 5 mm mirror aperture to guarantee a tilt-free out-of-plane translation (see Figure 3b). One single pantograph consists of six torsional springs (see Figure 3a,c): two springs arranged on the same axis and connected by stiff levers (SEM photographs of mechanical pre-deflected MEMS samples are presented in Figure 4). Due to the orthogonal–anisotropic elastic moduli of monocrystalline silicon, a design with four pantographs instead of three was chosen to be more robust to fabrication tolerances and less sensitive to parasitic mirror tilt. Details of the three different spring axes are shown in Figure 3 and Figure 5. In addition to the torsional springs, other mechanical structures are visible, used for limiting maximal out-of-plane translation.

The pantograph geometry was optimized to achieve (i) a lower frequency for an out-of-plane translation oscillation mode of 250 Hz and (ii) reduced viscous gas damping and demands on vacuum pressure using pantograph levers compared to previous MEMS. In general, the final MEMS design was developed within an iterative design process using finite element analysis (FEA) simulations of single and coupled physical domains with ANSYS^TM^ Multiphysics, as well as simulation of dynamic transients (e.g., voltage-dependent frequency–response curves of parametric resonance) using reduced-order models (ROMs).

#### 2.1.2. Reduction of Dynamic Mirror Deformation

**Figure 6:**
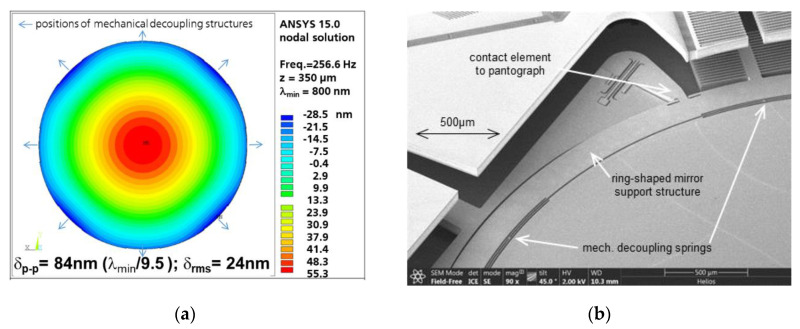
Reduction of dynamic mirror deformation: (**a**) FEA results of topology at 350 µm deflection; (**b**) SEM photograph of outer ring-shaped support structure with mechanical decoupling springs.

To reduce the dynamic mirror deformation of the 75 µm thick silicon mirror plate to the NIR-FTS specified value for dynamic deformation of *δ_pp_* ≤ 80 nm = 1/10 λ_min_, the best design compromise was found by reducing the resonance frequency of the used translation mode to ~250 Hz. The surface topology of the dynamically deformed mirror plate, occurring for a harmonic mirror oscillation of 256.6 Hz at a maximal *z*-deflection of 350 µm, is shown in Figure 6a. A satisfactory dynamic mirror deformation of *δ_pp_* = 84 nm = *λ_min_*/9.5 and *δ_RMS_* = 24 nm was simulated.

**Figure 7:**
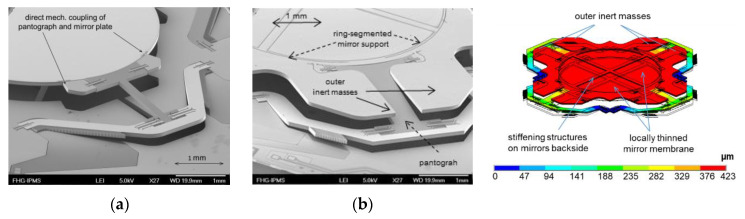
Further options to minimize the mirror deformation of pantograph MEMS: (**a**) initial design with direct coupling of pantographs and mirror plate; (**b**) conceptual design for dynamic self-compensation of dynamic mirror deformation by means of additional outer inert masses and locally thinned mirror membrane.

**Figure 8:**
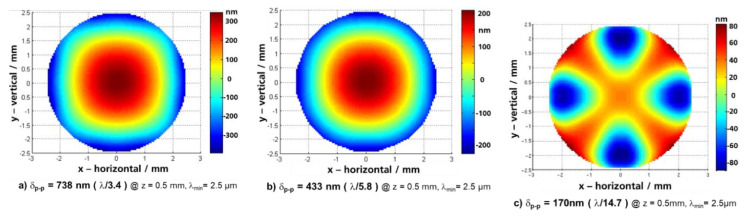
FEA results of minimization of mirror deformation of 5 mm pantograph mirrors of 75 µm thick c-Si; translatory MEMS designs were developed for MIR-FTS for 500 µm amplitude at 500 Hz and λ_min_ = 2.5 µm: (**a**) initial MEMS design with direct mechanical coupling of pantograph suspensions and mirror plate; (**b**) with ring-shaped pantograph support structure and mirror held by mechanical decoupling structure; (**c**) dynamic self-compensation by means of additional outer inert masses and locally thinned mirror membrane.

We have to mention here that the mirror deformation could not be minimized to the specified limit only by reducing the oscillation frequency. On the one hand, the MEMS device becomes mechanically fragile and sensitive to mechanical shocks (also crucial for MEMS fabrication). On the other hand, a direct mechanical coupling of pantograph suspension and mirror plate significantly deforms the mirror in addition to the dynamic forces caused by inertia (see Figure 7a and Figure 8a).

To avoid the additional deformation, the circular mirror aperture of 5 mm diameter is kept flexible by narrow radial springs within an outer ring-like support structure attached to the pantograph suspensions (see Figure 6b). The additional soft spring elements enable the mechanical decoupling of the mirror plate from pantograph suspension. This makes the dynamic deformation profile almost rotationally symmetric, like a free circular plate with translational oscillation in *z*.

In the past, several options to reduce dynamic mirror deformation for large-stroke translatory MEMS were investigated (results not yet published). We have to point out that the 75 µm SOI thickness of the mirror plate was not changeable due to the MEMS process AME75. Three variants of large-stroke pantograph MEMS designs were tested: (i) initial design with direct coupling of pantographs and mirror plate (see Figure 7a, [25]), (ii) ring-like support structure with mechanical decoupling of inner mirror (Figure 6b; variant used also within this work), and (iii) a conceptual design for dynamic self-compensation of mirror deformation by means of additional outer inert masses of a locally thinned mirror membrane (Figure 7b). The resulting dynamic mirror deformations of these variants are shown in Figure 8. For better comparison, all variants were simulated with 5 mm mirror diameter and identical operation point (MIR-FTS: 500 µm amplitude, 500 Hz, λ_min_ = 2.5 µm).

It is obvious for this study that the initial variant results in a poor mirror planarity of λ_min_/3.4 (see Figure 8a), whereas the variant with ring-shaped support reduces the mirror deformation by a factor of 1.7 (Figure 8b). A minimal dynamic deformation of only λ_min_/14.7 was simulated for the conceptual design with dynamic self-compensation (Figure 8c). On the other hand, the conceptual design results in higher technological complexity (local thinning of the mirror backside by deep reactive ion etching, DRIE), resulting also in higher risks for asymmetries, tilting, and sensitivity to mechanical shock.

Therefore, we used in this work the ring-shaped pantograph support structure and 250 Hz resonance frequency, resulting in a dynamic mirror deformation of *δ_pp_* = 84 nm = *λ_min_*/9.5, which is sufficient for the NIR-FTS applications (Figure 6).

#### 2.1.3. Modal Analysis

**Figure 9:**
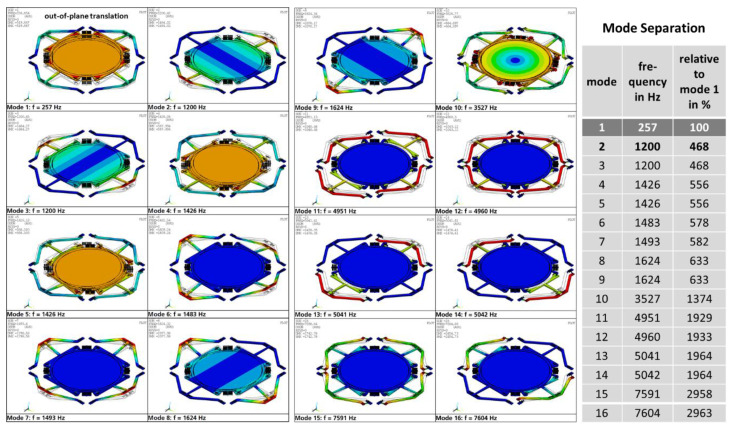
Results of FEA modal analysis: eigenmodes and separation to used translation mode 1 at 257 Hz.

The FEA results of the linear modal analysis are summarized in Figure 9 for the first to 16th eigenmodes. Here, a good mode separation of the used translation mode 1 at 257 Hz to the next higher parasitic (tilting) mode 2 at 1200 Hz is obvious. Higher modes are only of practical relevance during the wire-bonding process or when used in mechanically rough environments, if they can be excited by high-frequency external vibrations. To avoid parasitic excitations, integral multiples of mode 1 must avoided during the MEMS design, which is guaranteed for this translatory MEMS device.

#### 2.1.4. Electrostatic Comb Drives and Mechanical Reliability

**Figure 10:**
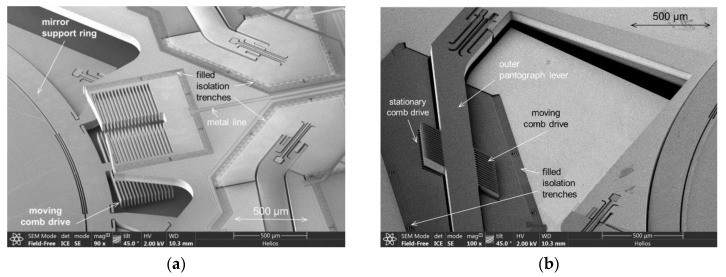
SEM photographs of electrostatic comb drives (visible are also the filled isolation trenches): (**a**) basic comb drives at ring-like support structure; (**b**) comb drive variant at pantograph levers.

This MEMS device is actuated by electrostatic comb drives, driven in parametric resonance [25,55]. Two versions of comb drive variants were investigated: (i) basic comb drives attached to the ring-like support structure (see Figure 3b and Figure 10a), and (ii) additional comb drives at the pantograph levers (Figure 4 and Figure 10b) to increase available frequency bandwidth at 350 µm amplitude. The results of this article are based on MEMS devices with basic comb variants only (see Figure 10a), using four comb drives symmetrically arranged at the support ring. Using ROM simulations of viscous gas damping [56] and modifying the model parameters to a reduced vacuum pressure, a driving voltage of 44.3 V was simulated to reach an amplitude of 350 µm at an assumed vacuum pressure of 10 Pa. This is slightly below the simulated electrostatic stability (pull-in) voltage of *U_pull-in_* = 46.5 V. Hence, a WLVP with <10 Pa inner vacuum pressure is required.

Concerning mechanical reliability, a mechanical stress of *σ_1_* = 0.5 GPa at a maximal deflection of 350 µm and *σ_1,eq_* = 1.47 GPa at 2500× *g* equivalent shock acceleration were simulated using nonlinear FEA simulations. Both stress values were below the design limit of ≤1.5 GPa, required for high mechanical reliability. To enhance the robustness to mechanical shocks, additional mechanical structures were designed, which are arranged parallel to the torsion springs (see Figure 5). In the event of mechanical contact due to mechanical shock, they cause the springs to stiffen and limit the out-of-plane translation. This stiffening concept was first demonstrated in [24] (see Figure 4 on pp. 6).

### 2.2. MEMS Device Wafer Fabrication

**Figure 11:**
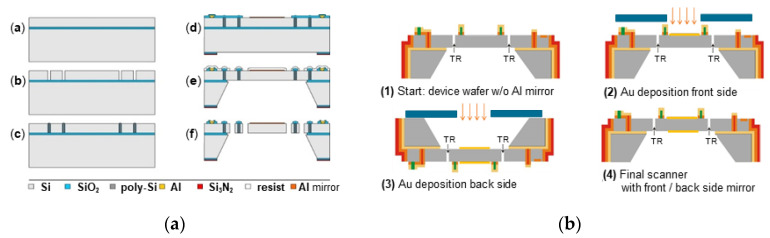
Fabrication of device wafer: (**a**) MEMS process AM75; (**b**) backend integration of Au coating.

The MOEMS devices were fabricated by the qualified IPMS process AME75, developed for electrostatic comb-driven micro scanning mirrors (Figure 11). They use 6” BSOI (bonded silicon-on-isolator) substrates with a 75 µm thick, highly p-doped SOI device layer, 1 µm buried oxide (BOX) layer, and 400 µm handling layer. Details of the MEMS process can be found, e.g., in [45]. For this work we have to point out the following specifics of the device wafer (DW) relevant for the MEMS WLVP:

- Use of field isolation trenches (formed from DRIE-etched open trenches by thermal oxidation and refilling with polysilicon) to electrically isolate areas of different electrical potential within the same SOI layer needed to define the comb drives (Figure 10),
- Use of AlSiCu metal lines for electrical signal transmission from the bond islands at outer chip frame to the inner comb drive actuator (no VIA (vertical interconnect access) exists),
- Use of a thin protected aluminum layer as standard optical coating,
- CMOS (complementary metal–oxide–semiconductor) compatibility of all inline processes due to restrictions caused by in-house CMOS processes for highly integrated micro mirror arrays [57].

The standard Al coating is not possible to meet the high reflectance of R > 95% required for the NIR-FTS. Therefore, we used Au for reflection coating and a symmetric coating design for thermal compensation to guarantee a small static mirror deformation of ≤λ_min_/10 after the WLVP process. Due to CMOS compatibility, in a backend process, identical Au coatings were deposited on the front and rear faces of the silicon mirrors using shadow masks for lateral patterning of the Au coatings.

### 2.3. Wafer-Level Vacuum Package of Optical MEMS

In the previous MIR-FTS development, a WLVP was not applicable due to (i) the need to use a ZnSe window, and (ii) the limitation to use an open trench isolation [25], because a field trench isolation was not available for 75 µm SOI at the time. In addition to the ZnSe window, this open trench insolation (needed also around bond islands located outside the vacuum cavity) prevented any hermetic vacuum sealing on the wafer level. In this work, the challenge for hermetic sealing of WLVPs results from the topology and roughness of the DW within the areas needed for the future bonding frames. These bonding areas are crossed by the metal lines required to contact the outer bond islands. Profilometer measurements showed a significant topology of ~1.8 μm (pp) [52] on these DW locations. Due to the significant surface topology, most bonding methods which could potentially be used for vacuum-packaging (see Table 2), such as metallic thermo-compression bonding, solid-liquid-inter-diffusion (SLID), eutectic AuSi bonding [39,44], and anodic bonding [46], are not applicable to our WLVP process.

**Figure 12:**
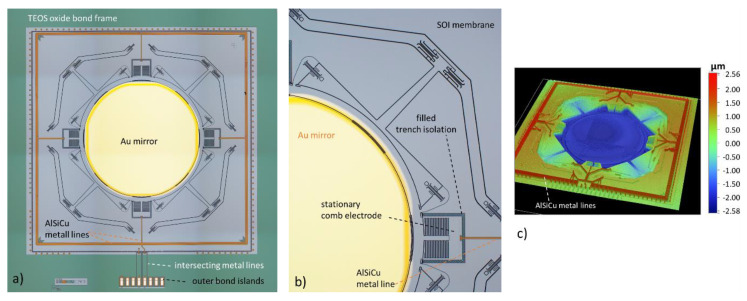
Translatory MEMS device after fabrication of device wafer: (**a**) chip photograph of MEMS chip without WLVP; (**b**) microscopic detail of pantograph suspension and comb drive; (**c**) MEMS chip topography measured with WLI (white light interferometry); metal lines add significant height profile.

For this work, glass-frit bonding [47,48,49,50,51,52] is the most reliable bonding method, allowing hermetic sealing over surface topologies several µm high. This bonding approach allows realizing hermetic electrical interconnects using existing metal lines without complex technological changes for the DW. Only the mask layout was designed to form a closed tetra-etyhl-ortho-silikat (TEOS) oxide frame on DW within the areas reserved for glass-frit bonding (see Figure 12a). First results on WLVP using glass-frit bonding for hermetic vacuum sealing of electrostatic resonant tilting MEMS scanners were reported in [52]. An inner vacuum pressure of 2–20 mbar (200–2000 Pa) was estimated without using a getter. This is insufficient for this translatory MEMS, requiring an internal pressure of at most 10 Pa. Hence, a thin film getter [58,59,60] is needed for this WLVP, using a highly efficient zirconium (Zr)-based thin-film getter from SAES Getters (SAES Getters S.p.A., Lainate (MI), Italy).

#### 2.3.1. Concept of WLVP for NIR-FTS

**Figure 13:**
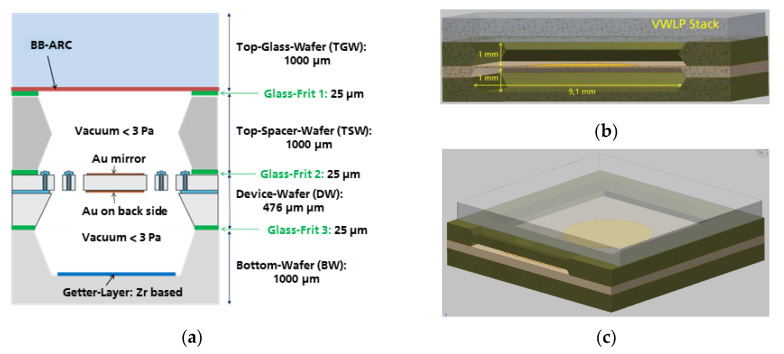
Schematic set-up of the MEMS WLVP optimized for NIR-FTS: (**a**) schematic cross-section of WLVP stack; total thickness of WLVP stack is 3476 μm + thicknesses of glass-frit layers; (**b**) overall dimensions of encapsulated cavity; (**c**) MEMS WLVP chip.

The schematic set-up of the optical MEMS wafer-level vacuum package (WLVP) is shown in Figure 13a. The WLVP consists of four pre-fabricated substrates, sequentially bonded by three glass-frit wafer bonding processes to form the final WLVP stack of 3476 μm total thickness in addition to 3× the layer thickness of the glass-frit bonding layer.

In addition to the 476 µm thick SOI device wafer (DW), containing all active (movable) MEMS structures, the WLVP stack consists of a 1000 µm thick top glass wafer (TGW, used as optical window), a 1000 µm thick (100) Si top spacer wafer (TSW), and a 1000 µm thick bottom (100) Si spacer wafer (BW). A double-sided polished borosilicate-glass wafer with high NIR transmission is used as the top glass wafer (TGW). For technological simplification of WLVP, an ordinary parallel configuration of the optical glass window was chosen instead of wedged optical windows. Parasitic etalon modulations on the measured NIR spectrum arising from multiple reflections between the TGW interior surface and the mirror surface were reduced by means of (i) a broadband antireflective coating (BB-ARC) deposited on the interior surface of the TGW, and (ii) a TSW of 1000 µm thickness, which always guarantees a minimum distance ≥650 µm between the TGW interior surface and the oscillating mirror, resulting in an etalon-free spectral range slightly smaller than a spectral resolution of ∆ν = 8 cm^−1^. The bottom wafer (BW), which finally hermetically seals the backside of the WLVP stack, contains a 600 µm deep cavity (TMAH (tetra-methyl-ammonium-hydroxide) etched) with a patterned Zr-based thin-film getter. For electrical characterization, the diced WLVP chips were glued and wire-bonded to a PCB (printed circuit board) using an additional 1 mm thick glass substrate on the backside of WLVP for simplified handling.

#### 2.3.2. Process-Flow for NIR-WLVP

**Figure 14:**
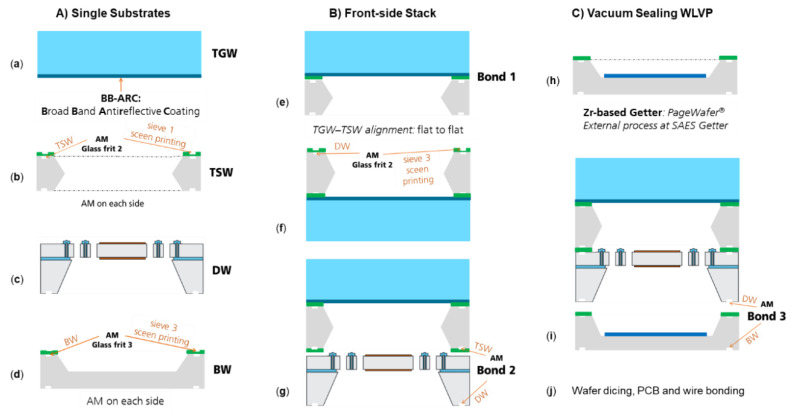
Schematic process flow of wafer-level vacuum package for translatory MEMS: (**a**) 1 mm thick borofloat-glass wafer with NIR BB-ARC on interior surface; (**b**) screen printing and pre-bake of first glass-frit layer on TSW (top spacer wafer) front-side; (**c**) device wafer (DW) with symmetric Au coating on Si mirror plate; (**d**) screen printing and pre-bake of third glass-frit layer on bottom wafer (BW) front-side; (**e**) first bonding of top glass wafer (TGW)–TSW stack using flat-to-flat alignment; (**f**) screen printing and pre-bake of second glass-frit layer on TSW backside; (**g**) second bonding of TGW–TSW–DW stack; (**h**) deposition of Zr-based thin-film getter, using the extern PageWafer process of SAES Getters; (**i**) third bonding for final hermetic vacuum sealing of WLVP.

The schematic process flow of the WLVP is shown in Figure 14.

**Figure 15:**
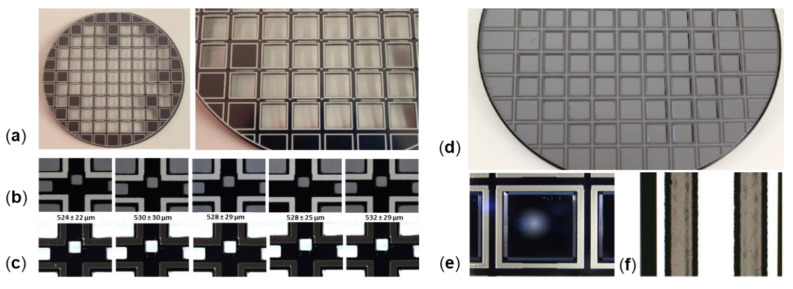
Screen-printed glass-frit bonding frames: (**a**) first glass-frit layer on TSW front-side; (**b**) after drying process at 120 °C; (**c**) after glazing process at 425 °C; (**d**) third glass-frit layer on BW alignment; (**e**) bond frame after drying at 120 °C; (**f**) after getter deposition, using PageWafer process of SAES Getters.

For fabrication of the top spacer wafer (TSW), a double-sided, polished, 1000 µm thick, (100)-oriented Si wafer was used. The free optical apertures were realized by TMAH wet etching simultaneously from either side (using a SiO_2_ hard mask). Then, a first, 20 µm thick glass-frit bond layer (Ferro FX-11 036) was screen-printed (patterned by the sieve) on the top spacer wafer. For all glass-frit bonding frames, a width of 490–550 µm was chosen to avoid defects during wafer dicing. After screen printing, the glass-frit layers were first dried at 120 °C, followed by a glazing process at 425 °C for hardening and out-gassing of the glass-frit frame before bonding (see Figure 15). This was followed by the first bonding process to bond the top glass wafer (TGW) and top spacer wafer (TSW) at 440 °C (equal to the glass temperature of the glass-frit layer). For simplicity, the bond interface is located on top of the BB-ARC (uniformly deposited on the inner side of the TGW). Here, the TGW and TSW were aligned flat to flat. Next, the second glass-frit bond layer was screen-printed on the opposite side of the top spacer wafer, using identical processes for drying and pre-baking. Then, the stack of TGW and TSW was glass-frit bonded to the front side of the device wafer (DW) using a slightly reduced bond temperature of 435 °C, in order not to influence the first bond interface.

The third glass-frit bond layer (used for final hermetic vacuum sealing of WLVP) was screen-printed on top of the BW before the getter deposition process (see Figure 15b). This was used in order to achieve a thermal decoupling of getter material from the high process temperature needed for glass-frit pre-bake. Otherwise the Zr-based getter layer would be completely activated and already saturated in the ambient atmosphere and would have no effect within the WLVP. The Zr-based thin-film getter was externally deposited, using the PageWafer^®^ process of SAES Getters.

The final hermetic vacuum sealing process consists of two main steps: (i) pre-heating to 200 °C for 2 h before contacting the wafer stack, still under vacuum pumping at process pressure, in order to degas the glass-frit layer and avoid early getter activation in step 1, and (ii) final hermetic vacuum sealing of the WLVP stack at 430 °C and final bonding pressure. Here, the getter material is activated during heating when temperature increases to *T* > 300 °C. In this study, we also investigated the influence of the vacuum process pressure (using 0.1, 1, and 25 Pa) on the inner vacuum pressure of WLVP.

**Figure 16:**
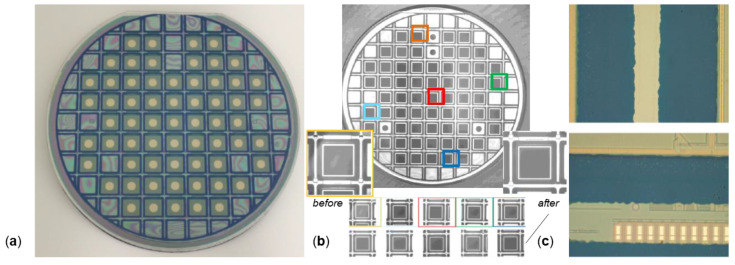
Final wafer-level vacuum package (WLVP) of translatory MEMS: (**a**) 6” wafer of MEMS WLVP before dicing; (**b**) infrared microscopic image of bond interface; details of bonding frame before and after third bonding process used for final hermetic vacuum sealing of WLVP; a good homogeneity of the bond is evident; (**c**) details of bond frame (bond islands are still encapsulated).

The WLVP bonding processes were performed using a bond aligner SÜSS BA8 (SÜSS MicroTec SE, Garching, Germany) and an SÜSS SB8 wafer bonder (SÜSS MicroTec SE, Garching, Germany). The final wafer level vacuum package (WLVP) of translatory MEMS is shown exemplarily in Figure 16 before wafer dicing. The details of infrared and microscopic images show a good homogeneity of the bonding frame. Finally, the 3476 µm thick WLVP stack was separated by wafer dicing into individual chips. Moreover, the bond pads were opened for wire bonding using a three-step sawing process. For characterization, the MEMS WLVP chips are mounted and wire-bonded onto a dedicated PCB.

## 3. Results and Discussion

### 3.1. Basic MEMS Characteristics without WLVP

**Figure 17:**
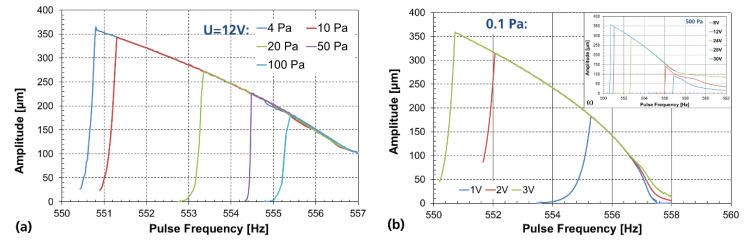
Frequency–amplitude characteristics (experiment without WLVP using external vacuum chamber): (**a**) varied pressure of 4–100 Pa @ 12 V; (**b**) voltage dependency at 0.1 Pa; (**c**, **insert**) at 500 Pa.

Initially, we studied the amplitude–frequency behavior depending on vacuum pressure and driving voltage using MEMS devices without WLVP. This is a commonly used method to estimate the vacuum influence on MEMS performance, e.g., [40,61], helpful to determine the minimum vacuum requirements for further WLVP development. Therefore, the MEMS samples were placed into a small vacuum chamber of 67 cm^3^ inner volume, using a vacuum turbo pump for external evacuation. The vacuum pressure inside the cavity could be varied from ambient pressure down to 0.1 Pa. The amplitude of translatory oscillation was measured through an optical window using a Michelson interferometer set-up with a helium–neon (He–Ne) laser operating at 632.8 nm wavelength. The MEMS devices are driven in parametric resonance, electrically excited with a pulsed driving voltage of 50% duty cycle and pulse frequency of twice the mechanical oscillation frequency. A frequency down-sweep is required to start the oscillation. Before this test, the stability (pull-in) voltage of *U_pull-in_* = 45 V was measured, limiting the maximal driving voltage. The dependence of the amplitude on pressure is exemplarily shown in Figure 17a for 12 V driving voltage.

It is obvious that a vacuum of below 4 Pa is needed to guarantee a stable parametric resonance oscillation at 575.5 Hz (pulse frequency) with 350 µm amplitude; this operation frequency is only 0.2 Hz above the instable resonance point [45,55]. The voltage dependence of the parametric resonance curves is shown for two relevant vacuum levels: at 0.1 Pa (target for this WLVP using getter, see Figure 17b) and 500 Pa (see Figure 17c, assuming a glass-frit bonded WLVP without getter, realized previously in [52]). To achieve the full amplitude of 350 µm, a driving voltage of 30 V is needed for 500 Pa, which can be significantly reduced in this study to 3 V at 0.1 Pa. It was observed for this translatory MEMS that scan amplitude cannot be set independently by driving voltage and frequency (see Figure 17). It is obvious that all parametric resonance curves lie directly on top of each another. They do not form a parametric family of curves (as is usually the case for torsional MEMS scanners [45]), i.e., there exist no amplitude–frequency curves that can be displaced relative to each other via the driving voltage. On the other hand, the increase in driving voltage only causes an increase in maximum scan amplitude, accompanied by a decreasing frequency of the (unstable) resonance point. This behavior (lack of additional parametric response curves, displaced by a parameterized driving voltage) is explained by (i) the small electrostatic actuation range in *z* of comb drives (see Figure 10a) and (ii) the degressive spring characteristic [62].

**Figure 18:**
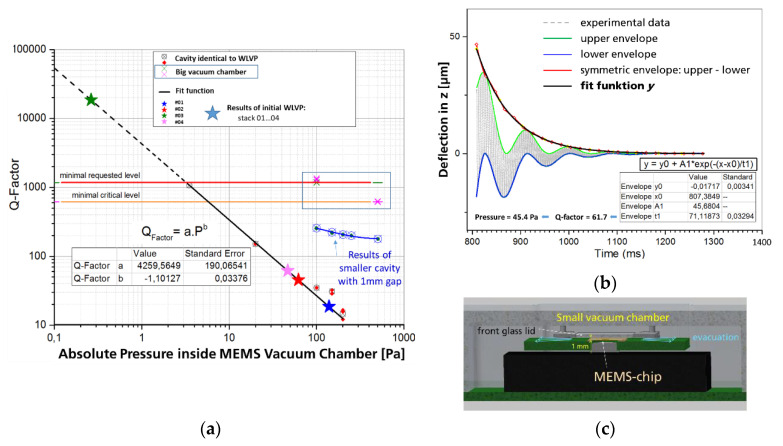
Experimental determination of minimum requirements for vacuum pressure inside the WLVP cavity: (**a**) results on vacuum pressure dependency of *Q* factor; obvious is the final calibration characteristic for determination of the vacuum pressure inside the WLVP; shown are also intermediate experimental results on the influence of cavity size; (**b**) exemplary result of determination of the *Q* factor from freely damped oscillation measured with laser vibrometer; (**c**) new set-up with small cavity and 1 mm gap.

Further experiments show a non-negligible influence of the volume of encapsulated vacuum cavity on the resulting pressure dependency of the MEMS characteristics (see Figure 18a). We modified the experimental set-up (i) to determine realistic values of the minimum vacuum requirements for the development of the WLVP, and (ii) to allow also an indirect measurement of the internal vacuum pressure inside the WLVP using a pressure-calibrated MEMS characteristic as a reference. Therefore, we encapsulated the MEMS within an additional cavity (similar in size and volume to the real WLVP), both evacuated inside the external vacuum chamber (see Figure 18c). We needed two iterations of this modified set-up to eliminate size effects. For reference, the pressure-dependent MEMS characteristics were first verified using the Michelson interferometer set-up. Finally, the vacuum pressure dependencies of scan amplitude and *Q* factor were measured with a laser vibrometer (Polytec MSA 500). Here, *Q* factors were calculated from the freely damped MEMS oscillation (see Figure 18b). We have to point out that this method is only applicable for large oscillation amplitudes >10 µm. Using a calibration of the internal pressure in terms of the measured *Q* factor, the minimum requirements for MEMS WLVP (defined for meeting the full 350 µm amplitude at ≤8 V) were determined as follows:

- maximal vacuum pressure: *p_max_* = 3.21 Pa,
- minimum *Q* factor: *Q_min_* = 1177,
- critical values (lower limits): *Q* = 600 and *p_max_* = 5.5 Pa.

### 3.2. Characteristics of Initial MEMS WLVP Run

In the initial WLVP fabrication run, four wafer stacks, i.e., (i) two stacks without getter and gold coating and (ii) two stacks with zirconium (Zr)-based getter and gold coating, were vacuum-bonded at 25 Pa process pressure. Using the calibrated characteristic of Q vs. vacuum pressure, it was possible to measure the inner vacuum pressure within the cavity of the WLVP.

#### 3.2.1. MEMS Characteristics with WLVP

For the two WLVP stacks without getter, an insufficiently high internal vacuum pressure of 139 Pa (Q = 18.6) and 62 Pa (Q = 45.3) was measured below the critical values. On the other hand, for the WLVP stacks with getter, a sufficiently high Q factor of 18,000 (equal to 0.25 Pa) was achieved for the WLVP stack 03. The second stack 04 failed (p = 46.8 Pa and Q = 61.7) for an unknown reason.

**Figure 19:**
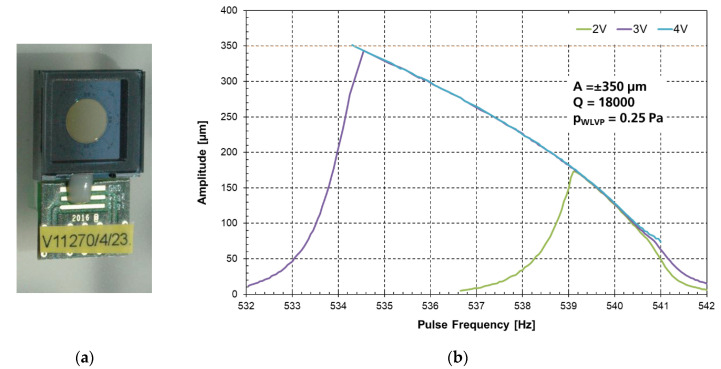
Experimental results of translatory MEMS with WLVP: (**a**) MEMS sample with WLVP; (**b**) frequency–amplitude characteristic in parametric resonance (sample of stack 03; initial WLVP run).

The frequency amplitude of a functional MEMS WLVP (sample of stack 03 measured with the Michelson interferometer set-up) is exemplarily shown in Figure 19b. At 0.25 Pa inner vacuum pressure, a driving voltage of 4 V is needed for an amplitude of 350 µm. The long-term stability of the inner vacuum was initially verified for WLVP stack 03 up to >10 years [54] using an ultrafine Ne vacuum leakage test [63], with an estimated mean time to getter saturation of 6.34 years. The long-term stability of the inner vacuum of stack 03 was experimentally verified after 32 months again, resulting in a vacuum of 0.47 Pa and a new estimated (residual) mean time-to-getter-saturation of 7.23 ± 1.54 years. These results clearly demonstrate the potential of the present WLVP strategy for a sufficiently long lifetime >10 years.

#### 3.2.2. Influence of Process Temperature on Optical Coating

**Figure 20:**
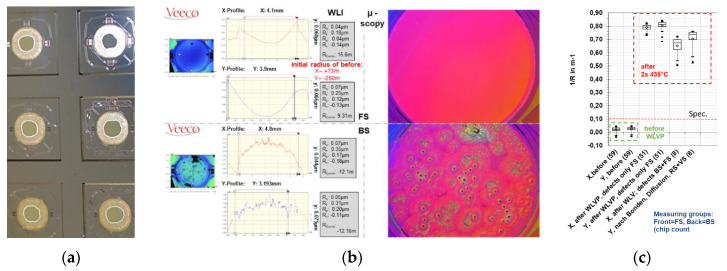
Experimental results on influence of process temperature on Au mirror coating: (**a**) defect images of mirror coating on wafer backside of stack 04 (before final vacuum bonding); (**b**) comparison of diffusion defects (white-light-interferometry (WLI), microscopy) on mirrors front- and backside; (**c**) comparison of static mirror deformation before and after the WLVP process.

During the initial WLVP process, the MEMS devices have to withstand two process steps at elevated process temperatures of 435 and 440 °C due to hermetic glass-frit bonding. These high process temperatures can be crucial for the static planarity of the Au-coated mirrors. To investigate the influence of process temperature on optical mirror coating, the static mirror deformation was measured for WLVP stacks 03 and 04 before and after the WLVP process using white-light interferometry (WLI). Different commercial Au coatings were used: Au-variant 1:10 nm Cr + 70 nm Au coatings for stack 04 (identical for the front- and backside) and Au-variant 2:7 nm Cr + 70 nm Au coatings (identical for the front- and back surfaces) for stack 03. The WLI results for stack 04 are summarized in Figure 20; significant defects caused by the diffusion of Au into the silicon substrate are obvious. These diffusion Au defects are less frequent at the front surface but affected 100% of the samples on the backside (see Figure 20a). In consequence, a clearly too large static mirror curvature of 1/*R* = 0.50–0.85 m^−1^ (equal to *δ_pp_* = 1.5–2.5 µm and compared to the small initial curvature of 1/*R* = 0.0014–0.0454 m^−1^ with *δ_pp_* = 4.5–142 nm static mirror deformation) was measured with WLI for stack 04. The results of stack 03 with Au coatings of variant 2 had fewer defects and a better performance, with final curvature 1/*R* = 0.13–0.19 m^−1^ (equal to *δ_pp_* = 395–440 nm) after WLVP process in comparison to the initial values in the range 1/*R* = −0.10 to −0.14 m^−1^ (*δ_pp_*= 309–440 nm).

#### 3.2.3. Discussion on Improvements of MEMS WLVP

The significant degradation of mirror planarity was caused by diffusion of gold into the silicon mirror plate, resulting in higher mechanical stresses. It also disturbed the original symmetry of the mirror coating required for temperature compensation. The higher defect density on the rear mirror surface can be explained by its higher thermal load in the bonding tool. As a consequence, the chromium adhesion layer could not safely prevent the Au diffusion. In order to prevent Au diffusion, we tested an additional, diffusion-tight barrier layer, which was deposited on the bare silicon mirror surface before the Au coatings were deposited. A 40 nm thick barrier layer of Al_2_O_3_ was homogenously deposited with atomic layer deposition (ALD) onto the entire DW. First, we simulated the influence of the process temperature on the (i) mirror planarity and (ii) the defect density of the Au coating after the WLVP process using MEMS dummy wafers (without electrical function). Two groups were tested (i) with and (ii) without Al_2_O_3_ diffusion barrier layer. We also compared the previously tested variants of Au coatings of two different commercial suppliers.

**Figure 21:**
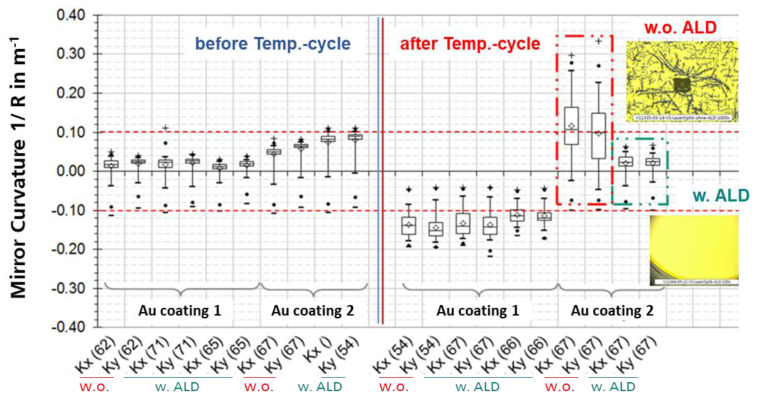
Improvement of the Au mirror coating and reduction of the influence of the process temperature on the Au mirror coating; experimental results of mirror planarity of simulated WLVP processes using MEMS dummy wafers, comparison of samples with and without diffusion barrier layer of 40 nm thick Al_2_O_3_ deposited by ALD. *Kx* and *Ky* denotes the curvature in *x-* and *y*-directions, respectively.

For the experimental simulation of the WLVP process, the samples were exposed to the same temperature cycles and thermal budget as in the real process. In Figure 21 the results on mirror curvature are displayed before and after the simulated WLVP process as boxplots for all tested variants. It is obvious that the Au coating variant 2 with an ALD barrier layer achieved the smallest mirror curvature of 1/R = 0.02 ± 0.015 m^−1^ corresponding to a static mirror deformation of *δ_pp_* = 55.1 ± 27.9 nm. Without the ALD barrier layer, small Au diffusion defects on the backside of the wafer result in a larger curvature and a broader variation range. In all samples with the Al_2_O_3_ diffusion barrier layer, no diffusion defects occurred after the simulated thermal process load.

**Figure 22:**
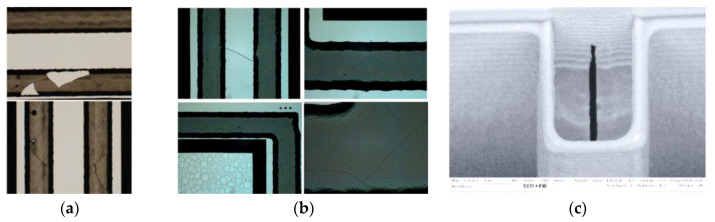
Defect images of potentially parasitic effects on yield and long-term stability of inner vacuum: microscopic images of (**a**) local cracks and spalling of BW glass-frit frame after getter deposition at SEAS; (**b**) small cracks inside glass-frit bonding frame of BW before getter deposition; (**c**) SEM photograph of open void inside filled trench isolation.

The reason for the poor vacuum of the defective WLVP stack 04 (with *p* = 46.8 Pa, although a Zr-based getter was used) was suspected to originate in local defects inside the glass-frit bonding frame. After external getter deposition at the SAES Getters, local defects of cracks and spalling were observed (see Figure 22a, bond frame before hermetic vacuum sealing), which could probably increase the leakage rate. The receiving inspection at SAES also showed small cracks within the previously defect-free glass-frit bond frames. The hypothesis of an increased leakage rate for WLVP stack 04 due to cracks was checked by means of an ultrafine neon vacuum leakage test [63].

However, the test showed a low leakage rate identical to the good samples. In other words, the glass-frit bond frames were hermetically sealed. The cause of the error had to be inside the vacuum cavity. By means of a comparative test using residual gas analysis (RGA) at SAES Getters, a high pressure of 4.4 mbar (440 Pa) and an unusual residual gas atmosphere was measured, containing 97% hydrogen and methane (inside the cavity) only for tested WLVP samples of the affected stack 04. This indicates an internal source of outgassing. Microscopic investigations and correlation with further SEM inspections revealed local defects of opened voids within the filled isolation trenches (see Figure 22c). The microscopic inspections found that 36.4% of all device wafers of this study were affected by these defects, observed also in wafer stack 04 but not for stack 03. It is suspected that these voids contained polymer residuals from the DRIE passivation process. In the improved WLVP process, the 40 nm thick Al_2_O_3_ barrier layer, deposited on the entire DW by atomic layer deposition (ALD), may help to encapsulate these void defects. Afterward, failure (observed at stack 04) no longer occurred.

To reduce the risks for hermeticity of WLVP, the final glass-frit bonding process used for hermetic vacuum sealing of WLVP was changed. To avoid any cracks or spalling inside the final glass-frit bonding frame (caused during the external getter deposition), we decoupled the getter deposition on the BW. For this purpose, we screen-printed the final glass-frit bonding frame (used for hermetic vacuum sealing of WLVP) on the backside of the DW. The thermal influence of this additional third glass-frit bonding process (affecting the mirror planarity) was tested to be tolerable.

### 3.3. Final Characteristics of Improved MEMS WLVP

**Figure 23:**
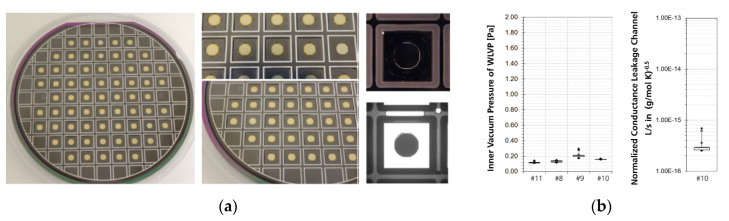
Final results of WLVP: (**a**) process change of third glass-frit bond frame on backside of DW; (**b**) boxplot of inner vacuum pressure for WLVP stacks (left) and normalized conductance of leakage channel measured for WLVP stack #10.

Finally, four WLVP stacks were fabricated with the improved process (exchange of third glass-frit layer to DW backside, see Figure 23a), using also a reduced vacuum process pressure to enhance the effective getter capacity of WLVP. Two samples each were vacuum-bonded at 5 Pa (#8, #9) and 0.1 Pa (#10, #11). The inner vacuum pressure was measured for individual chips with a laser scanning vibrometer (Polytec MSA500) using the calibration method shown in Figure 18. In comparison to the initial results (see Figure 19b: 25 Pa process pressure, resulting in 0.25 Pa of WLVP), we achieved a slightly reduced median vacuum pressure of 0.155 ± 0.019 Pa. The boxplot diagram of inner vacuum pressure is shown in Figure 23b. We only found a neglectable dependency on vacuum process pressure, resulting in 0.170 ± 0.013 Pa inside WLVP for 5 Pa and 0.114 ± 0.025 Pa for 0.1 Pa. Furthermore, higher *Q* factors were measured, typically in the range of *Q* = 38,600–48,500.

**Figure 24:**
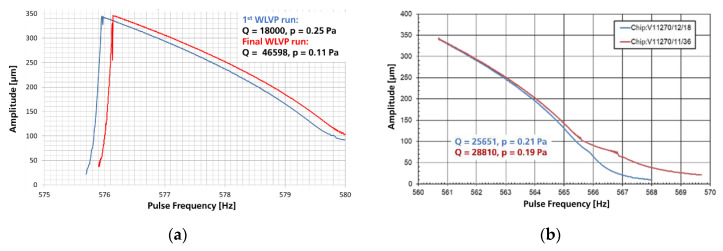
Frequency amplitude characteristics in parametric resonance at 4 V: (**a**) influence of *Q* factor on frequency characteristic, shown are WLVP MEMS of the initial and final WLVP run; (**b**) exemplary comparison of two WLVP MEMS devices (from different wafers); good reproducibility is obvious.

The influence of a higher *Q* factor on the resulting frequency characteristic is exemplarily shown in Figure 24a for two samples of the initial (*Q* = 18,000) and final (*Q* = 46,598) WLVP run. The rise of the frequency response curves is fairly identical. No steeper characteristic (as anticipated) is visible for the sample with higher *Q* value. On the contrary, it is somewhat flatter due to the used higher driving voltage of 8 V (preferred in the meantime for FTS integration). Furthermore, in Figure 24b, a good reproducibility of frequency characteristics is shown. From both diagrams, a robust WLVP process window for inner vacuum pressure and frequency characteristic is obvious.

Furthermore, the time until complete getter saturation (afterward, the inner vacuum pressure starts to increase) was estimated using several time-shifted vibrometer-based control measurements of the internal pressure using individual chips of stack #10. Therefore, a normalized conductance of the leakage channel to the inner WLVP cavity of 0.166 cm^3^ volume was calculated for stack #10 (Figure 23b) as follows:

(1) $$\tilde{L}=\left(2.5\;10^{-16}\;\pm 2.3\;10^{-18}\right)\;\frac{L}{s}\sqrt{\frac{g}{mol.K}}@\;20\;{}^{\circ}C.$$

Using this value and the specification of the used PageWafer^®^ thin-film getter, we calculated the time until complete getter saturation (after which the internal pressure of WLVP starts to increase). This calculation is based on the assumption of a temporarily constant conductance of the leak channel and without internal sources of outgassing. A mean time to getter saturation of 7.91 ± 0.08 years was calculated for stack #10.

**Figure 25:**
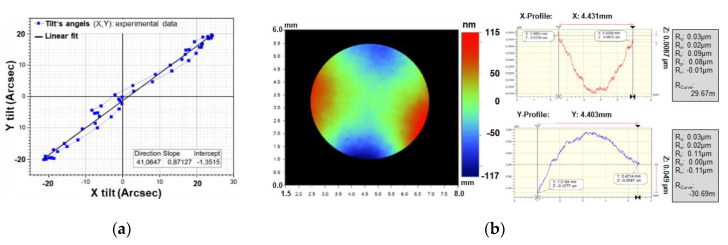
Exemplary results of (**a**) parasitic mirror tilt within a full translational oscillation of 350 µm amplitude, and (**b**) static mirror deformation of the Au-coated mirror plate after WLVP process.

The parasitic tilt angles in *x* and *y*, occurring during a translatory oscillation in *z* with 350 µm amplitude, were also measured for individual samples with laser scanning vibrometry (Polytec MSA 500). We observed parasitic tilt angles in the range of 20–80 arcsec, where an average of 60.2 ± 15.6 arcsec was achieved. A result of a sample with a small parasitic mirror tilt of ±20 arcsec is exemplarily shown in Figure 25a.

The resulting static mirror deformation of the final WLVP (measured with white-light interferometry) is exemplary shown in Figure 25b. A mean static mirror deformation of *δ_pp_x_* = 100.1 nm and *δ_pp___y_* = −87.3 nm was measured in the *x-* and *y*-directions. While the absolute values of the curvatures along the *x-* and *y*-directions are within the permissible limits, they now have opposite signs, in contrast to the previous results of Figure 21. This saddle deformation arises from an incorrect alignment of the shadow masks used for the patterning of front and back side Au coatings.

## 4. Conclusions

In this article, we presented a wafer-level vacuum-packaged (WLVP) translatory MEMS actuator developed for a compact NIR-FT spectrometer with high SNR >1000 in the spectral bandwidth of 1200–2500 nm. For this purpose, two objectives had to be solved which were not available with the current state of the art: (i) design of a large-stroke pantograph MEMS device with minimized dynamic mirror deformation of ≤80 nm and (ii) development of a glass-frit-based optical WLVP (to be compatible to the fixed MEMS process), avoiding degradation of the optical mirror quality at high process temperatures.

The large-stroke resonant MEMS design uses a fully symmetrical four-pantograph mirror suspension, avoiding problems with tilting and parasitic modes. To minimize dynamic mirror deformation, the best design compromise was found in (a) reduction of the resonance frequency of out-of-plane translation to <270 Hz and (b) mechanical decoupling of the 5 mm mirror plate from the pantograph suspension using a ring-shaped support structure with additional radial decoupling springs. The use of additional stiffening structures at the mirror backside [64] was avoided to simplify the MEMS process. This MEMS design approach results in a sufficiently small dynamic mirror deformation of *δ_pp_* = 84 nm = λ_min_/9.5 at 350 µm amplitude and 267 Hz out-of-plane translation, driven electrostatically in parametric resonance. Due to significant gas damping, the MEMS device has to be operated in vacuum. In a first step, we experimentally studied the influence of vacuum pressure and cavity size on the MEMS behavior. The minimum requirements of ≤3.21 Pa and *Q* = 1177 inside the WLVP cavity of 0.166 cm^3^ size were determined by laser vibrometer experiments. From these experiments, we also developed an experimentally verified calibration model for the *Q*-factor-based determination of the inner vacuum pressure inside the MEMS WLVP.

The challenge for the hermetic sealing of this MEMS is the significant surface topology ≥2 µm caused by the AlSiCu metal lines crossing the sealing area. For the hermetic sealing of NIR-WLVP, we selected the glass-frit bonding to be best suited and technologically compatible with our in-house MEMS scanner process AME75, resulting in lower development efforts. In contrast to alternative bonding approaches (see Table 2), the highly ductile 25 µm thick glass-frit bond layer safely seals the WLVP hermetically and simultaneously forms embedded lateral signal feedthroughs to outer bond islands. On the other hand, glass-frit bonding requires high process temperatures of 430–440 °C, which the MEMS device has to withstand without compromising its optical performance. In comparison to eutectic alloy bonding (e.g., Au_0.80_Sn_0.20_ with a eutectic temperature of 280 °C [39]), glass-frit-based WLVP also requires a broader sealing frame, resulting in a larger chip size and higher costs. In our case, the 530 µm wide sealing frame is fully acceptable compared to the large overall MEMS chip size of 12.4 × 12.4 mm^2^. The NIR-FTS optimized WLVP was developed in two iterations to investigate failure modes and optimize reliability. For a high reflectance ≥95% in NIR, we had to use Au for optical coating. To enable thermal compensation of the stresses induced by the bonding processes, we used a symmetric coating design [65], depositing identical Au coatings on the front- and backside of the mirror. A small static mirror deformation of ≤100 nm was achieved after the WLVP process. To guarantee a long-term stable inner vacuum pressure of 0.2 Pa, we applied a Zr-based thin-film getter using the external PageWafer process from SAES Getters. In the initial WLVP run, we achieved 0.25 Pa inner vacuum pressure of the WLVP and a *Q factor* of 18,000 at 25 Pa process pressure, resulting in a driving voltage of only 4 V to meet the required amplitude of 350 µm. After 32 months, the remaining mean time-to-getter saturation was estimated to be 7.2 ± 1.5 years, which demonstrates the potential of the WLVP for a sufficiently long lifetime >10 years.

On the other hand, several serious problems were observed for the original WLVP concept [54]. Initially, the 70 nm thick Au coatings with 7–10 nm Cr adhesion layer were deposited directly onto the silicon mirror plate. After the WLVP process, significant mirror defects and unacceptable large mirror deformations caused by Au diffusion into silicon were observed. Via ALD deposition of an additional 40 nm thick Al_2_O_3_ diffusion barrier layer (which was homogeneously deposited on the entire MEMS device wafer prior to Au deposition), this problem could be completely eliminated. In addition to fixing the Au–Si reaction (not prevented by a Cr layer only), the conformed Al_2_O_3_ layer is also advantageous to encapsulate inner sources of out-gassing contained within the DW (e.g., voids of the filled trenches). In this work, another potential reliability problem for the final glass-frit bond was observed. In the initial WLVP run, the final glass-frit bond layer (containing also the thin-film getter) was deposited on the BW before getter deposition using the PageWafer process from SAES Getters. After getter deposition, small cracks or spallings were observed inside the final glass-frit bonding frame. To avoid this potential risk for WLVP hermeticity, we finally switched the third glass-frit layer from BW to DW backside in order to decouple the final hermetic vacuum sealing from the getter deposition.

In the final WLVP process, the inner vacuum pressure was reduced to 0.15 ± 0.02 Pa and higher *Q* factors 38,600–48,500 were measured due to a reduced process pressure of 0.1–5 Pa. However, it was shown that the process pressure has a minor influence on the internal vacuum pressure. The achieved inner vacuum pressure is comparable to the state-of-the-art WLVP of MOEMS [42], but avoids the thermal degradation of mirror planarity observed for glass-frit bonded micro scanners in [52,53]. Finally, static mirror deformations of ≤100 nm were measured for WLVP samples with symmetric Au coatings and additional 40 nm thick Al_2_O_3_ diffusion barrier layers. The mean time-to-getter saturation was estimated to be 7.9 ± 0.1 years. The residual dynamic tilt angle (which occurs during a full translational oscillation with 350 µm amplitude) was measured to be in the range of 20–80 arcsec. For NIR-FTS system integration, MEMS devices with small tilt must be selected to guarantee an SNR >1000 in the spectral bandwidth 1200–2500 nm. Finally, we compared the performance of this NIR-FTS-specific pantograph MEMS device (350 µm amplitude, resonant operation at 267 Hz, dynamic mirror deformation of *δ_pp_* = 84 nm) with the latest state of the art for MEMS-based NIR-FTS. In [64], a MEMS-based NIR-FTS with 7 nm spectral resolution was reported using a translatory MEMS with 3 mm mirror diameter, driven electrostatically in resonance at 265.5 Hz, resulting in an amplitude of 125 µm in normal ambient conditions. At 125 µm amplitude, a parasitic tilt of 2.2/1000° and a mirror deformation of 100 nm were measured. Compared to [64], our pantograph MEMS has a higher optical throughput and a 2.8-foldhigher amplitude. Hence, it has the potential for increased spectral resolution and higher SNR.

## 5. Summary and Outlook

Although monolithic, highly miniaturized MEMS-based NIR-FTSs exist today, we follow a classical optical FT instrumentation using a resonant MEMS with precise out-of-plane translatory oscillation of a 5 mm diameter mirror for optical path-length modulation. Our advantages are a higher optical throughput and resolution in comparison to highly miniaturized systems, as well as mechanical robustness and insensitivity to vibration and mechanical shock, compared to conventional FTS mirror drives. The new vacuum WL-packaged translatory MEMS devices are very promising for compact FTSs, potentially allowing to replace expensive and complex conventional mirror drives. The versatility, high acquisition rate, and robustness of an MOEMS-based FTS makes it ideal for process control and applications in harsh environments (e.g., surveillance of fast chemical reactions). This potentially enables a new family of compact FT analyzers for the NIR spectral region *λ* = 800–2500 nm with a spectral resolution of ≤15 cm^−1^, 500 scans/s, and SNR > 1000 within an acquisition time of 1 s (with co-addition of spectra). It should lead to a sensitive, reliable, and easy-to-use stand-alone NIR-FT spectrometer qualified for industrial applications in harsh environments, e.g., applied for ad hoc inspection of food quality or environmental parameters. The results of the final system integration into a miniaturized FT-NIR spectrometer (using selected MEMS devices with minimal parasitic tilt) will be published elsewhere. For further developments of NIR-FTS systems, new developments should also be considered [64,66,67,68].

Compared to alternative WLVPs, the glass-frit bonding process offers long-term hermeticity along with good sealing properties and tolerates the topography of the joining partners by a planarizing effect of the softened glass-frit during the bonding process. In parallel, conductive tracks or metallic lead-throughs for device signaling can be covered within the softened glass, preventing the need for processing complex and obviously more expensive vertical device signaling [48,49], with new questions arising related to hermeticity and device design and compatibility. Additionally, glass-frit bonding offers process flexibility due to a wide variety of suitable substrates including CMOS compatibility [50]. By application-related benchmarking of glass-frit bonding with alternative WLVP, glass-frit bonding can offer a good compromise of challenges, advantages, and disadvantages with respect to the specific features of the final application [39], required tooling, and the involved manufacturing processes. Additionally, glass-frit bonding still offers potential in terms of reducing the bonding temperature below (already available) 380 °C with ongoing glass-frit material development, substituting lead-based with lead-free glass-frit materials and using alternative or improved glass-frit deposition methods. In order to minimize outgassing behavior, the tuning of the thermal conditioning of the glass frit and the involved bonding procedures could be investigated, along with the reduction of the size and volume of the printed glass-frit bonding interface and its influence on the resulting hermeticity and reliability of the device.

## Figures and Tables

**Table 1:** Specification of NIR-FTS and boundary conditions for MEMS device.

FTS Specification	Value	MEMS Boundary Conditions	Value
Mirror aperture	5 mm	Maximal mechanical stress *s*_1_	≤1.5 GPa
Resonance frequency	250–300 Hz	Maximal shock acceleration	2500 g
Mechanical amplitude	350 µm	Mirror deformation δ_pp_	80 nm
Spectral range [λ_min,_ λ_max_]	800–2500 nm	Reflectance of mirror	≥95%
Spectral resolution ∆ν	≤15 cm^−1^	Parasitic tilt angle	20”
Lifetime	≥10 years	Vacuum pressure in WLVP	≤1 Pa

**Table 2:** Qualitative comparison of wafer bonding approaches for applicability on MOEMS-WLVP.

	Bonding Approach	Bonding Temperature (°C)	Topo-Graphy Tolerance	Main Advantage	Main Disadvantage	Ref.
**Traditional approaches**	Anodic Direct Glass-frit	300–450 >800 430–450	Low Very low High	Good for silicon–glass - Excellent hermetic sealing, possibility of incorporating metallic feedthroughs	High process voltage Poor CMOS compatibility Large seal area	[46] [46] [46,47,48]
**Metal Bonding Approaches**	Eutectic SLID (solid-liquid-inter-diffusion) Metal–to-metal	>200 >200 >200	Medium Medium Low	Ductile seal Re-melting temperature is greater than bonding temperature Mature process	Complex deposition Lack of ductility in seal No collapse layer to absorb topology	[39,46] [39,46] [46]
**Adhesive**	BCB	>150	High	Ductile seal	Non-hermetic	[46]
